# Fluid tolerance assessed by lung ultrasound and effect of crystalloid expansion on extravascular lung water in critically ill children with cancer

**DOI:** 10.1016/j.jped.2025.101456

**Published:** 2025-10-14

**Authors:** Bruno S. Camargo, Orlei R. de Araujo, Dafne Cardoso B. da Silva

**Affiliations:** Universidade Federal de São Paulo (UNIFESP), Instituto de Oncologia Pediátrica, Grupo de Apoio ao Adolescente e à Criança com Câncer (GRAACC), Pediatric Intensive Care, São Paulo, SP, Brazil

**Keywords:** Extravascular lung water, Critical illness, Cancer care unit, Plasma volume, Diagnostic ultrasound

## Abstract

**Objectives:**

To investigate the effect of a bolus of saline on extravascular lung water (EVLW), and to analyze correlations of EVLW with outcome.

**Methods:**

Prospective cohort. Patients received 10 mL/kg of saline (bolus). Central venous blood gas analysis and echocardiography measurements were performed before and after. Lung ultrasound was performed with 12 measurements, repeated after expansion. Statistics included paired *t*-test and logistic regression models. The magnitude of the effect was assessed using Cohen's test.

**Results:**

88 measurements were made on 83 patients. There was a response to volume in 48 measurements (54.5 %). 28 patients (33.7 %) had signs of shock, and 60 were on mechanical ventilation (MV, 72.2 %). In 68 paired measurements, there was an increase in B-lines after expansion (77.2 %). The mean number of B-lines pre- was 1.42 (SD 1.12) per intercostal space, increasing to 1.71 (SD 1.17) post-expansion (*p* < 0.001, Cohen's d 0.97). The mean pre-expansion cardiac index was 3.43 L/min/m2 (SD 1.05), increasing to 3.87 (SD 1.21, *p* < 0.001, Cohen's *d* = 0.8). In a multivariate model, lactate and mean pre-expansion B-lines were independent predictors of death in ICU: pre-B-lines, Odds ratio 1.87, *p* = 0.012; pre-lactate OR 1.59, *p* = 0.012. The same was observed with post-volume B-lines, in a model which also included MV: post-volume B-lines, OR 1.87, *p* = 0.01; post-volume lactate, OR 1.51, *p* = 0.03; MV, OR 4.32, *p* = 0.041.

**Conclusions:**

Most patients showed signs of fluid intolerance, with increased EVLW. EVLW assessed by ultrasound is a predictor of mortality.

## Introduction

Intravenous fluid administration is a cornerstone in the resuscitation of critically ill children, particularly in conditions such as septic shock, hypovolemia, and dehydration. Despite its essential role in restoring intravascular volume and improving perfusion, excessive or inadequate fluid administration can lead to adverse effects, including interstitial pulmonary edema. Extravascular lung water (EVLW) accumulation is a significant concern in critically ill patients,^1^^,^^2^ as it can compromise gas exchange, increase respiratory distress, and prolong the need for mechanical ventilation. Fluid accumulation in critically ill patients is strongly associated with increased morbidity and mortality.^3^

Crystalloids are the most commonly used fluids for fluid resuscitation due to their availability, low cost, and ease of administration. However, their potential to contribute to pulmonary edema remains a subject of debate, especially in children with impaired capillary permeability or cardiac dysfunction.^1^^,^^3^^,^^4^ In large volumes, they can exacerbate capillary leak and increase hydrostatic pressure, leading to fluid accumulation in the interstitial and alveolar spaces of the lung.^2^^,^^4^ Identifying early markers of fluid-induced pulmonary edema is crucial to optimizing fluid therapy and preventing complications.^5^ Lung ultrasound has emerged as a valuable tool for noninvasive assessment of EVLW. The presence and quantification of B-lines, hyperechoic artifacts extending from the pleura into the depth of the image caused by fluid-filled interstitial spaces, have been proposed as a surrogate marker for pulmonary congestion.^1^^,^^4^^,^^6^ Ultrasound allows real-time monitoring of lung fluid status and is particularly useful for guiding fluid management in critically ill children. There are limited data on its impact on pediatric oncology patients, who may have additional risk factors for altered fluid dynamics.

This study aimed to investigate the impact of intravenous saline administration on EVLW, quantifying B-lines, and analyzing possible correlations with ICU outcome (discharge or death).

## Methods

The study was conducted in the oncology intensive care unit (ICU) of a pediatric cancer center in São Paulo, Brazil. The Research Ethics Committee of the Federal University of São Paulo approved the protocol (Certificate of Submission of Ethical Appraisal number 68803323.9.0000.5505, date of approval July 19, 2023. Title: Echocardiographic evaluation of fluid response in children admitted to the ICU). The study was conducted in accordance with Brazilian ethical regulations (resolution 196/96 of the National Health Council) and with the Helsinki Declaration of 1975. Informed consent was obtained from all guardians of the patients.

The study design was a prospective interventional cohort study involving patients admitted to the ICU from July 2023 to March 2025. Inclusion criteria were age under 18 years, diagnosis of oncological disease and critical condition, presence of a central venous catheter for blood collection, and free and informed consent of the guardians. Patients who did not have viable transthoracic echocardiographic windows, with congenital heart disease, significant intracardiac shunts or coarctation of the aorta, left ventricular dysfunction with ejection fraction < 50 % on functional echocardiography at ICU admission, or with severe arrhythmias were excluded. Patients with signs of pulmonary congestion in the initial assessment of extravascular lung water, with a mean of more than two B lines per intercostal space, or the presence of confluent B lines, considered contraindications to fluid administration, were also excluded.

Study patients received a 10 ml/kg (maximum 500 ml) saline bolus, programmed to be infused in a maximum of 30 min, and were reassessed post-infusion (30 – 60 min). Baseline demographic data were collected. Central venous blood gas analysis, echocardiography, and lung ultrasound measurements were performed before and after fluid volume. The percentage of fluid overload in the last 24 h was calculated as:^7^

%offluidoverload=(sumofdaily(fluidin−fluidout)/admissionweight)×100

The inferior vena cava collapsibility index was calculated as:^8^

IVCCI=(inferiorvenacavamaximumdiameter−minimumdiameter/maximumdiameter)×00

Transthoracic echocardiography was performed by one of the authors with ultrasound experience in children in intensive care, using a device available in the unit (Sonosite Turbo®). Lung ultrasound was performed with a linear transducer with a bandwidth of 13–6 MHz, with 12 measurements, 6 on the right (intercostal spaces 3rd, 4th, and 5th in 2 lines, midaxillary and anterior) and the same on the left. The assessment of B-lines was repeated after expansion to assess the impact on EVLW. Often, a comprehensive protocol of 28 sectors in the anterolateral chest is recommended for the quantitative assessment of B-lines. However, previous research has demonstrated a strong positive correlation between EVLW and simplified B-line scores obtained from chest examinations limited to 4 or 8 sectors. However, to date, comparison of different scanning protocols is scarce.^6^^,^^9^^,^^10^ In the present study, the authors chose to perform the count in 12 intercostal spaces to minimize patient manipulation, and this was also the reason for not taking the exam in duplicate with another examiner. Echocardiography for fluid responsiveness was performed using a 5–1 MHz cardiac transducer. Patients were considered to be volume responsive if an increase of > 15 % in cardiac index was observed.^11^

Descriptive statistics included measures of central dispersion (means and standard deviations - SD), in addition to absolute and relative frequencies. To compare the means of paired measurements, the authors used the paired T test. For correlations, the Spearman test. The magnitude of the effect, represented by Cohen's d, was used to describe the standardized mean difference in the effects of volume administration on EVLW. The way to interpret Cohen's d is as follows: very small effect (d: < 0.2), small effect (d: 0.2 to 0.5), moderate (*d* > 0.5 < 0.8), and large (*d* ≥ 0.8).^12^ Bivariate logistic models were made with the variables of central venous blood gas analysis, mean number of B-lines, serum electrolytes, lactate levels in central venous blood, use of vasoactive drugs, mechanical ventilation, cardiac index, hemoglobin, age and PRISM IV score, for the outcomes “ICU discharge” or “death”. Variables with p-values < 0.1 were included in multivariate models. Statistical analyses were performed using the software Jamovi version 2.6 and R: A language and environment for statistical computing.

## Results

During the study period, the authors had 786 admissions. Eighty-eight measurements were made on 83 patients who met the inclusion and exclusion criteria. Measurements in repeated patients were made on different ICU admissions.

Clinical and demographic data are presented in Table 1.

**Table 1:** Demographic data and general characteristics of patients.

Data		
Age (months, median, IQR)	69	39–100.5
Female (N, %)	37	44.5
**Cancer diagnoses**	**N**	%
Acute lymphoblastic leukemia	7	8.4 %
Acute myeloid leukemia	6	7.2 %
Neuroblastoma	7	8.4 %
Medulloblastoma	9	10.8 %
Wilms tumor	2	2.4 %
Burkitt's leukemia	3	3.6 %
Hodgkin's Lymphoma	1	1.2 %
Non-Hodgkin's Lymphoma	3	3.6 %
Teratoid rhabdoid tumor	5	6.0 %
Glioma	5	6.0 %
Other central nervous system tumors	15	18.1 %
Ewing's sarcoma	4	4.8 %
Others	16	19.3 %
**Immediate causes of admission (N, %)**		
Septic shock	19	22.9 %
Post-operative of neurosurgery/orthopedics/pediatric surgery	18	21.7 %
Acute respiratory failure	11	13.3 %
Hypovolemic shock	2	2.4 %
Other types of shock	7	8.4 %
Seizures	4	4.8 %
Upper gastrointestinal bleeding	3	3.6 %
Decreased level of consciousness	3	3.6 %
Sepsis without shock	3	3.6 %
Acute obstructive abdomen	2	2.4 %
Hyperleukocytosis	2	2.4 %
Intracranial hypertension	2	2.4 %
Other diagnoses	10	12.0 %
Hematopoietic Stem Cell transplantation (HSCT)	19	22.9 %
PRISM IV (mean, SD)	11.1	7.7
Vasoactive drugs	20	24.1 %
Mechanical ventilation	57	68.6 %
Neutropenia (< 500 cells/mm^3^)	28	33.7 %
Lymphopenia (< 500 cells/mm^3^)	41	49.4 %
Recent chemotherapy (< 30 days)	49	59.0 %
Induction	46	55.4 %
Maintenance	3	3.6 %

IQR, interquartile range 25–75; SD, standard deviation.

**Fig. 1:**
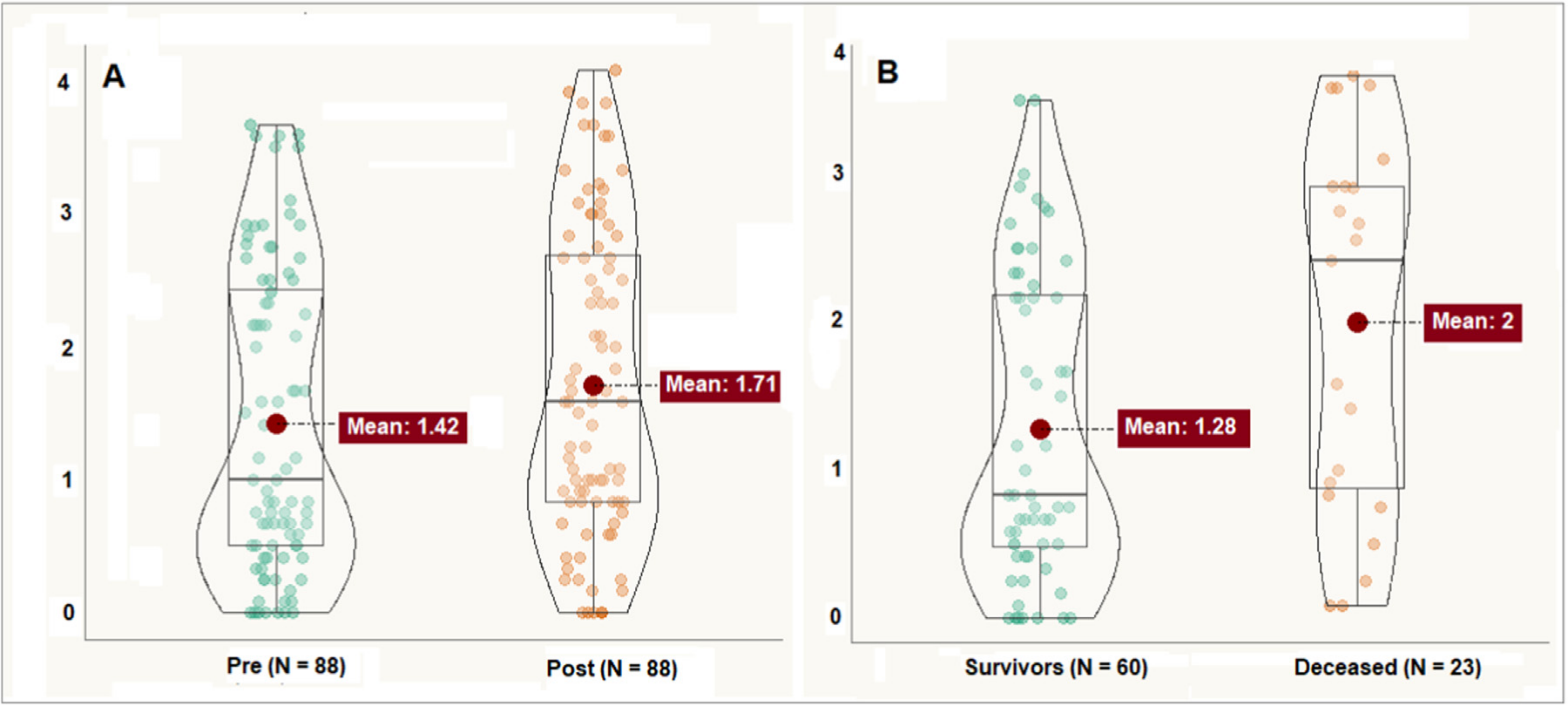
Violin boxplots showing the differences between the means of B lines per intercostal space pre and post 10 mL/kg volume bolus (panel A, *p* < 0.001), and between survivors and deceased (panel B, *p* = 0.02).

The median infusion time was 20 min (IQR: 15–21). The median infusion time was 20 min (IQR: 15–21). Ten patients were receiving concomitant epinephrine with a mean dose of 0.13 mg/kg/minute (SD: 0.09), and seven were receiving norepinephrine (mean dose of 0.16 mg/kg/minute (SD: 0.15). Three patients were receiving milrinone 0.5 mg/kg/minute, and concomitant use of norepinephrine and epinephrine occurred in 5 cases. In 48 measurements (54.5 %), the authors observed a response to volume expansion. The mean pre-expansion cardiac index was 3.43 L/min/m^2^ (SD 1.05), increasing to 3.87 (SD 1.21) after volume administration (*p* < 0.001, Cohen's *d* = 0.8). In 68 paired measurements, there was an increase in B-lines after volume expansion (77.2 %). The mean number of B-lines before volume expansion was 1.42 (SD 1.12) per intercostal space, increasing to 1.71 (SD 1.17) after expansion (Figure 1 panel A, *p* < 0.001). The magnitude of the effect showed a Cohen's d of 0.97.

Stratifying for patients on mechanical ventilation (75 %), the increase was also significant (mean pre: 1.46 (SD 1.17), post 1.76 (SD 1.2), *p* ≤ 0.001), with Cohen's *d* = 1.1. In those out of ventilation, there was also a significant change (mean pre: 1.36 (SD 1.05), post 1.61 (SD 1.12), *p* < 0.001), with Cohen's *d* = 0.79. It was also significant when considering patients who did not respond to volume (pre mean: 1.57 (SD 1.24), post 1.86 (SD 1.24), *p* < 0.001, Cohen's *d* = 1.2), and for those who responded, with a smaller effect size (pre mean: 1.31 (SD 1.07), post 1.59 (SD 1.1), *p* < 0.001, Cohen's *d* = 0.84). For patients with signs of shock, the increase was also significant (pre mean: 1.59 (SD 1.17), post 1.82 (SD 1.2), *p* < 0.001), with Cohen's *d* = 0.81. In another subgroup, that of patients with recent chemotherapy (< 30 days), there was also a difference in means (pre mean: 1.56 (SD 1.15), post mean: 1.67 (SD 1.17), *p* < 0.001, Cohen's *d* = 0.88. In the subgroup of patients with solid tumors, the pre mean was 1.12 (SD 1.02), and the post mean was 1.42 (SD 1.12), with *p* < 0.001 and Cohen's *d* = 1.02. It was not possible to evaluate the leukemia subgroup due to the small sample.

There were no significant effects on the other blood gas parameters, lactate, hemoglobin, and electrolytes.

There were 23 deaths (27.7 %), and the authors observed a difference between the means of pre-volume B-lines between patients who survived (mean 1.28, SD 1.04) and those who died (mean 2.00, SD 1.24, *p* = 0.008; Figure 1, panel B).

Among the 23 deaths in this sample, 20 were due to multiple organ failure, and three were due to the progression of oncological disease.

The significant bivariate and multivariate logistic models for the outcome “death in PICU” are shown in Table 2. Lactate and mean B-lines were independent predictors in the multivariate model, pre- and post-expansion. Mechanical ventilation was also an independent predictor, in a model that included mean B-lines and lactate post-expansion. Partial pressure of carbon dioxide (PCO_2_) correlated with the mean B-lines (Spearman's rho 0.46, *p* < 0.001) and was not included in the multivariate model. Other parameters of blood gas analysis, serum electrolytes, use of vasoactive drugs, cardiac index, hemoglobin, age and PRISM IV score, and the difference between the mean B-lines post- and pre-volume expansion were not predictors of death in the ICU.

**Table 2:** Bivariate and multivariate logistic models for the outcome “death in PICU”.

Bivariate logistic models - Pre-volume expansion	Estimates	Standard error	p	Odds ratio	Lower limit (95 % CI)	Upper limit
Mean B-lines	0.58	0.23	0.011	1.79	1.14	2.79
Mechanical ventilation	1.42	0.674	0.035	4.14	1.11	15.52
Lactate	0.43	0.17	0.011	1.54	1.10	2.14
PCO_2_	0.06	0.02	0.016	1.06	1.01	1.11
**Post-volume**						
Mean B-lines	0.59	0.23	0.008	1.81	1.16	2.82
Lactate	0.32	0.15	0.029	1.38	1.03	1.85
PCO_2_	0.06	0.02	0.026	1.06	1.01	1.11
**Multivariate logistic models - Pre-volume expansion**						
Mechanical ventilation	1.32	0.71	0.063	3.74	0.93	15.02
Mean B-lines	0.62	0.25	0.012	1.87	1.14	3.04
Lactate	0.46	0.20	0.020	1.59	1.07	2.35
**Post-volume**						
Mechanical ventilation	1.46	0.72	0.041	4.32	1.06	17.58
Mean B-lines	0.63	0.24	0.010	1.87	1.16	3.02
Lactate	0.42	0.19	0.030	1.51	1.04	2.20

The mean percentage of fluid overload in the 24 h before infusion was 0.55 % (SD: 3.5 %); in the bivariate logistic model, the percentage was not a predictor of the outcome “death in the ICU” (OR 0.89, *p* = 0.12), and was not included in the multivariate models. The same occurred with the inferior vena cava collapsibility index (mean 33.7 %, SD 17.8 %, OR 1.008, *p* = 0.58).

In the subgroup of patients with recent chemotherapy (< 30 days, *N* = 49, with 15 deaths), the only significant variable in the bivariate logistic model was mechanical ventilation (Odds ratio 14, 95 % CI 1.7–118). The wide confidence interval demonstrates the inadequate sample size for regression analysis in the subgroups. It was not possible to analyze the leukemia subgroup, with only 16 cases. In the solid tumor subgroup, there were 10 deaths in 55 cases, and the regression revealed no predictive variables, which can also be attributed to the low sample.

## Discussion

The authors observed a significant increase in the number of B lines after expansion with crystalloid solution at a volume of 10 mL/kg. These findings corroborate the existing literature, which indicates that volume expansion may not be without risks related to pulmonary congestion.^2^^,^^13^ A systematic review by Gan et al. found that only 40–69 % of critically ill children respond positively to volume administration.^14^ Fluid overload is closely associated with an increase in mechanical ventilation time, prolonged hospital stay, incidence of acute kidney injury, and a significant increase in morbidity and mortality.^15^^,^^16^^,^^17^^,^^18^

Virtually all patients receive varying amounts of fluid therapy during an episode of critical illness. There is consensus that fluids should be administered early and directed toward appropriate physiologic outcomes, but they have a narrow therapeutic index.^19^^,^^20^ In healthy individuals, 85 % of an infused crystalloid bolus is redistributed to the interstitial space after 4 h.^21^ In critically ill patients with endothelial injury and capillary leak, <5 % of a fluid bolus remains in the intravascular space after 90 min.^22^ The concept of fluid tolerance, which has emerged in recent years, can be expressed as the degree to which an individual tolerates fluid administration without organ dysfunction. It lies on the continuum between volume responsiveness and fluid overload.^16^ Organ dysfunction should be preceded by signs of venous congestion, and in this regard, point-of-care ultrasound can provide identifiable warning signs during the resuscitation process, allowing for more rational strategies. In 2005, Agricola et al. found a good correlation between the number of B-lines on lung ultrasound and EVLW in postoperative cardiac surgery patients. Their data demonstrated, for the first time, a correlation between lung ultrasound and invasive measurements of EVLW.^23^ The presence of fluid disrupts the normal air-fluid interface in the lung, allowing sound waves to reverberate and create B lines.^10^ Extravascular lung water represents the amount of cellular and extracellular fluid in the interstitial and alveolar spaces, and its increase is an important pathophysiological pattern of hydrostatic pulmonary edema. In adult patients, the EVLW value measured by the transpulmonary thermodilution method is associated with mortality in critically ill patients and is significantly higher in non-survivors than in survivors.^24^ A randomized, controlled trial conducted in neonates with septic shock revealed that ultrasound-guided fluid resuscitation resulted in reduced length of neonatal ICU stay, decreased length of hospital stay, and lower volume of fluids administered.^25^ The present study showed that the B-lines measurement method may be useful in identifying patients at higher risk of death, providing a window to guide therapy.

Cancer patients typically receive large volumes of fluid for hyper-hydration and chemotherapy. Systemic chemotherapeutics contact the endothelium before diffusing into the tissues, with abundant evidence of decreased endothelial function, reduced nitric oxide, and endothelial activation. When activated, endothelial cells acquire characteristics that activate procoagulant, inflammatory, and apoptotic mediators, leading to fluid leakage into the interstitial spaces. Capillary leak syndrome is a critical complication of hematopoietic stem cell transplantation.^26^^,^^27^ 22.9 % of the patients underwent HSCT, which can also lead to fluid overload by means of infusions of blood products, antibiotics, and parenteral nutrition. Transplant complications such as veno-occlusive syndrome and acute kidney injury increase the risk of volume overload. These particularities make critically ill children with cancer very susceptible to volume overload. In the study by Raymakers-Janssen et al., the percentage of volume overload at the start of continuous renal replacement therapy differed significantly between survivors and non-survivors (3.5 % versus 13.6 %), with almost 50 % of non-survivors experiencing weight gain of 10 to 20 %.^28^ “Fluid creep” (the fluids administered as a vehicle for drugs, to maintain patency of vascular catheters, and to flush venous lines) can be responsible for 60 % of the water administered to critically ill children and can also account for most sodium and chloride (56 and 58 %).^29^ Measures to restrict fluid creep, early use of diuretics, and indication of renal replacement therapy could theoretically reduce mortality in critically ill cancer patients when lung ultrasound indicates increased EVLW.

There is a widespread belief that volume-responsive patients do not develop venous congestion.^30^ However, even in these patients, fluid administration may be deleterious, depending on the clinical context. In this study, the authors observed that in the volume-responsive group, there was also a significant increase in B lines after expansion, although with a smaller magnitude of effect than in the non-responder group. It is important to emphasize that intervention through a fluid bolus did not appear to be harmful, since the difference between the pre- and post-mean B-lines was not predictive of the outcome of death, and the pre- and post-odds ratios were very similar.

The present study has several limitations, the most obvious being that it was a single-center study that included a heterogeneous and limited population of patients, whether or not on mechanical ventilation. The small sample size does not allow for the analysis of subgroups, such as septic shock, PARDS, HSCT, and leukemia. However, it showed significant effects of a single volume bolus, demonstrating that the use of lung ultrasound is a valuable tool in assessing changes in lung water volume over time, allowing rapid monitoring. It is a noninvasive, radiation-free, and accessible technique, which makes it an appropriate and competent method for bedside diagnosis among children in the ICU or emergency department setting.

Monitoring fluid tolerance by lung ultrasound has been shown to be a useful tool for monitoring EVLW after volume expansions. Most of the studied patients showed signs of fluid intolerance, with increased EVLW. EVLW assessed by ultrasound is a predictor of mortality in critically ill children with cancer. In the critically ill pediatric oncology patients in this study, EVLW assessed by ultrasound was associated with mortality. Due to the small sample, these results cannot be generalized to all subgroups, requiring external validation in multicenter studies with a larger sample and subgroup analysis.

## Financial support used for the study

Only institutional.

## Financial disclosures

None to declare.

## Data availability statement

The data that support the findings of this study are available from the corresponding author.

## Conflicts of interest

The authors declare no conflicts of interest.

## References

[1] Picano E., Pellikka P.A. (2016). Ultrasound of extravascular lung water: a new standard for pulmonary congestion. Eur Heart J.

[2] Jozwiak M., Teboul J.L., Monnet X. (2015). Extravascular lung water in critical care: recent advances and clinical applications. Ann Intensive Care.

[3] Alobaidi R., Basu R.K., DeCaen A., Joffe A.R., Lequier L., Pannu N. (2020). Fluid accumulation in critically ill children. Crit Care Med.

[4] Zanza C., Saglietti F., Tesauro M., Longhitano Y., Savioli G., Balzanelli M.G. (2023). Cardiogenic pulmonary edema in Emergency medicine. Adv Respir Med.

[5] Allinovi M., Saleem M., Romagnani P., Nazerian P., Hayes W. (2017). Lung ultrasound: a novel technique for detecting fluid overload in children on dialysis. Nephrol Dial Transplant.

[6] Picano E., Frassi F., Agricola E., Gligorova S., Gargani L., Mottola G. (2006). Ultrasound lung comets: a clinically useful sign of extravascular lung water. J Am Soc Echocardiogr.

[7] Selewski D.T., Cornell T.T., Lombel R.M., Blatt N.B., Han Y.Y., Mottes T. (2011). Weight-based determination of fluid overload status and mortality in pediatric intensive care unit patients requiring continuous renal replacement therapy. Intens Care Med.

[8] Garcia R.U., Meert K.L., Safa R., Aggarwal S. (2021). Inferior Vena Cava collapsibility index to assess Central venous pressure in perioperative period following cardiac surgery in children. Pediatr Cardiol.

[9] Enghard P., Rademacher S., Nee J., Hasper D., Engert U., Jörres A. (2015). Simplified lung ultrasound protocol shows excellent prediction of extravascular lung water in ventilated intensive care patients. Crit Care.

[10] Mayr U., Lukas M., Habenicht L., Wiessner J., Heilmaier M., Ulrich J. (2022). B-Lines scores derived from lung ultrasound provide accurate prediction of extravascular lung water index: an observational study in critically ill patients. J Intens Care Med.

[11] Boyd J.H., Sirounis D., Maizel J., Slama M. (2016). Echocardiography as a guide for fluid management. Crit Care.

[12] Lakens D. (2013). Calculating and reporting effect sizes to facilitate cumulative science: a practical primer for *t*-tests and ANOVAs. Front Psychol.

[13] Long E., O'Brien A., Duke T., Oakley E., Babl F.E., Pediatric Research in Emergency Departments International Collaborative (2019). Effect of fluid bolus therapy on Extravascular Lung Water measured by Lung Ultrasound in children with a presumptive clinical diagnosis of sepsis. J Ultras Med.

[14] Gan H., Cannesson M., Chandler J.R., Ansermino J.M. (2013). Predicting fluid responsiveness in children: a systematic review. Anesth Analg.

[15] Alepuz F.E., Ruz A.D., Santana J.D., Cebrian C.G., Encarnacion J., Sanz M.H. (2024). Fluid responsiveness in pediatrics: an unsolved challenge. Front Anesthesiol.

[16] Kattan E., Castro R., Miralles-Aguiar F., Hernández G., Rola P. (2022). The emerging concept of fluid tolerance: a position paper. J Crit Care.

[17] De Backer D., Aissaoui N., Cecconi M., Chew M.S., Denault A., Hajjar L. (2022). How can assessing hemodynamics help to assess volume status?. Intens Care Med.

[18] Maitland K., Kiguli S., Opoka R.O., Engoru C., Olupot-Olupot P. (2011). Mortality after fluid bolus in African children with severe infection. N Engl J Med.

[19] Bagshaw S.M., Brophy P.D., Cruz D., Ronco C. (2008). Fluid balance as a biomarker: impact of fluid overload on outcome in critically ill patients with acute kidney injury. Crit Care.

[20] Malbrain M.L., Marik P.E., Witters I., Cordemans C., Kirkpatrick A.W., Roberts D.J. (2014). Fluid overload, de-resuscitation, and outcomes in critically ill or injured patients: a systematic review with suggestions for clinical practice. Anaesthesiol Intens Ther.

[21] Chowdhury A.H., Cox E.F., Francis S.T., Lobo D.N. (2012). A randomised, controlled, double-blind crossover study on the effects of 2-L infusions of 0.9% saline and plasma-lyte(R) 148 on renal blood flow velocity and renal cortical tissue perfusion in healthy volunteers. Ann Surg.

[22] Sánchez M., Jiménez-Lendínez M., Cidoncha M., Asensio M.J., Herrerot E., Collado A. (2011). Comparison of fluid compartments and fluid responsiveness in septic and non-septic patients. Anaesth Intens Care.

[23] Agricola E., Bove T., Oppizzi M., Marino G., Zangrillo A., Margonato A. (2005). Ultrasound comet—Tail images”: a marker of pulmonary edema. A comparative study with wedge pressure and extravascular lung water. Chest.

[24] Gavelli F., Shi R., Teboul J.L., Azzolina D., Mercado P., Jozwiak M. (2022). Extravascular lung water levels are associated with mortality: a systematic review and meta-analysis. Crit Care.

[25] Huang D., You C., Mai X., Li L., Meng Q., Liang Z. (2024). Lung ultrasound-guided fluid resuscitation in neonatal septic shock: a randomized controlled trial. Eur J Pediatr.

[26] Terwoord J.D., Beyer A.M., Gutterman D.D. (2022). Endothelial dysfunction as a complication of anti-cancer therapy. Pharmacol Ther.

[27] Xue S., Chen J., Shi Y., Zhang L., Chen M., Sun H. (2025). Severe late onset capillary leak syndrome post allo-HSCT successfully treated by bevacizumab: a case report. Front Med (Lausanne).

[28] Raymakers-Janssen P.A., Lilien M.R., Tibboel D., Kneyber M.C., Dijkstra S., van Woensel J.B. (2019). SKIC (Dutch Collaborative PICU Research Network). Epidemiology and outcome of critically ill pediatric cancer and hematopoietic stem cell transplant patients requiring continuous renal replacement therapy: a retrospective nationwide cohort study. Crit Care Med.

[29] Langer T., D'Oria V., Spolidoro G.C., Chidini G., Scalia Catenacci S. (2020). Fluid therapy in mechanically ventilated critically ill children: the sodium, chloride and water burden of fluid creep. BMC Pediatr.

[30] Muñoz F., Born P., Bruna M., Ulloa R., González C., Philp V. (2024). Coexistence of a fluid responsive state and venous congestion signals in critically ill patients: a multicenter observational proof-of-concept study. Crit Care.

